# Exercise as a multiscale recalibration of stress-related homeostatic balance

**DOI:** 10.3389/fnins.2026.1801865

**Published:** 2026-05-11

**Authors:** Yan Chen, Xin Qiu, Guoxin Ni, Jie Shao, Fan Yang

**Affiliations:** 1 School of Sport Medicine and Rehabilitation, Beijing Sport University, Beijing, China; 2 Guangdong Provincial Key Laboratory of Brain Connectome and Behavior, The Brain Cognition and Brain Disease Institute, Shenzhen Institutes of Advanced Technology, Chinese Academy of Sciences, Shenzhen, China; 3 Department of Rehabilitation Medicine, The First Affiliated Hospital of Xiamen University, School of Medicine, Xiamen University, Xiamen, China

**Keywords:** brain circuits, brain-periphery communication, exercise, homeostasis, stress

## Abstract

Chronic stress disrupts homeostasis in the brain and body, leading to anxiety, depression, and cardiovascular and metabolic dysfunction. Although exercise can counter these effects, the mechanisms are scattered across fields and not yet integrated. This review proposes a multi-scale framework. Exercise is not only stress-relieving; it is also a controllable challenge that can recalibrate the system when repeated bouts are matched by sufficient recovery and bioenergetic support. We propose that repeated exercise engages a stress response–adaptation–recovery cycle, in which peripheral signals from skeletal muscles, liver, adipose tissue and gut convey body metabolic state to the brain and are consolidated into durable plasticity only when mitochondrial capacity, substrate availability, and redox balance permit recovery. These signals pass through the blood-brain barrier and engage plasticity switches, including neurotrophic signals, epigenetic modification and metabolic coupling, thus stabilizing the neural circuits of threat appraisal, reward processing and contextual memory. By integrating these dimensions, we clarify how exercise can transform short-term physical stress into lasting resilience and provide direction for future research.

## Graphical Abstract

**Figure:**
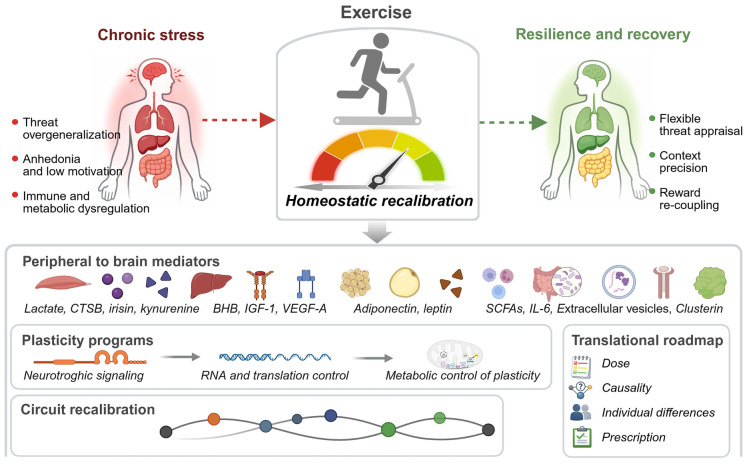

## 1 Introduction

Stress is fundamentally a biological necessity: a coordinated neuroendocrine and autonomic nervous system response to maintain stability in the face of challenges (McEwen, 1998). However, in modern society, this adaptive response can become maladaptive. Epidemiological data from the World Health Organization show that exposure to trauma and chronic stress is high, which contributes to a global crisis of anxiety and depressive disorders (World Health Organization, 2017; Anxiety disorders, n.d.; Depressive disorder (depression), n.d.). Clinically, these conditions are often accompanied by metabolic and immune dysfunction, suggesting that there is a systemic loss of homeostatic regulation of the brain and body.

It is also useful to distinguish acute from chronic stress. Acute stress is time-limited and recruits rapid autonomic and hypothalamic–pituitary–adrenal (HPA)-axis responses that can transiently enhance vigilance and action readiness. Chronic stress reflects repeated or sustained activation, impaired feedback regulation, and prolonged glucocorticoid exposure, with accumulating effects on prefrontal control, amygdala reactivity, hippocampal contextual processing, and peripheral metabolism. In this sense, the relationship between stress load and adaptation is not linear: moderate, well-bounded challenge may support adaptive performance, whereas insufficient or excessive arousal can impair it, broadly consistent with the Yerkes–Dodson principle (Herman et al., 2016; Godoy et al., 2018).

Exercise can help close this gap. Paradoxically, exercise itself can also act as a stressor. Vigorous exercise increases cortisol, activates the sympathetic nervous system, and mobilizes immune signaling (Geng et al., 2025). In many human contexts, and in voluntary-running paradigms, exercise is more controllable, time-limited, and predictable than chronic psychological stress; however, some externally imposed animal paradigms, such as treadmill running, can themselves recruit stress-related responses. Whether this challenge yields adaptation or instability depends on boundary conditions. Synaptic remodeling, neurogenesis, and epigenetic updating are energetically costly processes that require sufficient mitochondrial oxidative capacity, redox balance, substrate availability, and recovery time. Recent research suggests that chronic stress is directly linked to mitochondrial dysfunction, as repeated activation of the respond–adapt–recover cycle during stress coping can lead to an exhausted state that is accompanied by reduced mitochondrial oxidative capacity and impaired oxidative phosphorylation activity (Tippairote et al., 2026). We therefore view exercise-induced recalibration as a stress response–adaptation–recovery process rather than as signaling activation alone. This makes exercise a controllable challenge that can train the body’s stress-response systems. Many studies show that planned exercise can reduce depressive and anxiety-like symptoms, but the underlying mechanisms remain fragmented across fields (Stonerock et al., 2015; Noetel et al., 2024). Neuroscientists map circuits, molecular biologists track proteins, and physiologists measure blood metabolites, often within separate disciplinary frames. This disciplinary fragmentation motivates the present review, which seeks to integrate these levels into a unified mechanistic framework.

Unless otherwise stated, the circuit and molecular-level causal evidence discussed below derives primarily from rodent models, whereas the human literature provides the strongest support for clinical efficacy, systems-level neuroimaging correlates, and translational network-level convergence. Against this background of fragmented evidence across neuroscience, physiology, and clinical research, this review aims to integrate these levels within a structured multiscale framework of exercise-induced stress adaptation. We hypothesize that exercise restores stress-related homeostasis via a multiscale mechanism. As the stress-related emotional disorders are directly caused by the dysfunction of stressor-coping neural network. First, we examine the circuit-level outcomes, specifically how repeated training reshapes the networks that govern threat appraisal, contextual processing and reward, by strengthening prefrontal regulation and dampening amygdala hyperactivity. Next, we examine the molecular substrates that underpin these circuit shifts, explaining how plasticity programs such as brain-derived neurotrophic factor (BDNF) signaling, m^6^A epitranscriptomic regulation and histone lactylation (a post-translational modification that links lactate metabolism to gene regulation) stabilize synaptic adaptations that counter stress-related changes such as heightened anxiety and fear. Finally, we trace the peripheral origins of these signals, exploring how mediators produced by muscles, liver, and gut cross physiological barriers to link systemic metabolic states with central nervous system adaptation.

It should also be noted that the current mechanistic literature is weighted toward aerobic or endurance paradigms, especially voluntary wheel running and treadmill training in rodents. Resistance training also shows clinical benefit for depressive and anxiety symptoms, but its circuit-level and molecular mechanisms remain less well characterized (Gordon et al., 2017; Noetel et al., 2024). Accordingly, the present review emphasizes aerobic mechanistic evidence while highlighting the need for more comparative work across exercise modalities.

## 2 A multi-timescale framework for exercise-induced neuroplasticity and recovery

To understand exercise, it is important to consider the role of time. The benefits of physical activity stem from the integration of fast and slow processes, not a single molecule. The first phase is the acute phase, which can last from minutes to hours. Once exercise begins, the brain quickly receives a variety of neuromodulators, such as dopamine, serotonin and norepinephrine, along with a rise in endogenous cannabinoids and metabolic substrates (such as lactate). These signals can act as real-time state signals to change the arousal state and mood while preparing for changes in synapses. The key distinction is that exercise modulates the state of synapses through regulatory homeostatic mechanisms, such as adjusting signal-to-noise ratios and inhibitory tone, rather than simply increasing overall neural excitability.

The second stage is chronic, which lasts from days to weeks: through repetition, these short-term changes are consolidated. Across days to weeks, repeated exercise can consolidate transient neuromodulatory signals into more stable changes in synaptic efficacy, inhibitory control, trophic support, and, in selected regions such as the hippocampus, structural remodeling and adult neurogenesis. Exercise does not merely boost brain activity; it restores the signal-to-noise ratio, enabling the brain to distinguish true threats from background noise.

A third phase is recovery and consolidation. In the hours to days after each exercise bout, mitochondrial throughput, substrate replenishment, redox control, and sleep or behavioral recovery determine whether acute state signals are converted into stable synaptic remodeling and longer-term behavioral change (Vargas-Mendoza et al., 2021). In this sense, pathways such as BDNF–Tropomyosin-related kinase B (TrkB) signaling, mechanistic target of rapamycin (mTOR)-dependent translation, m^6^A remodeling, and lactylation are contingent on bioenergetic throughput rather than independent of it. If recovery is insufficient or energy reserves are limited, the same challenges may increase stress burden rather than resilience.

In this review, recalibration refers to a durable shift in stress-related operating ranges rather than a transient post-exercise state. Operationally, it includes more appropriate HPA-axis reactivity and recovery, improved circuit signal-to-noise and excitation–inhibition balance, more adaptive plasticity thresholds in threat, context, and reward circuits, and sufficient metabolic reserve to maintain these changes across repeated exercise–recovery cycles.

## 3 Circuit-level mechanisms: recalibrating threat appraisal, context processing, and reward

**FIGURE 1:**
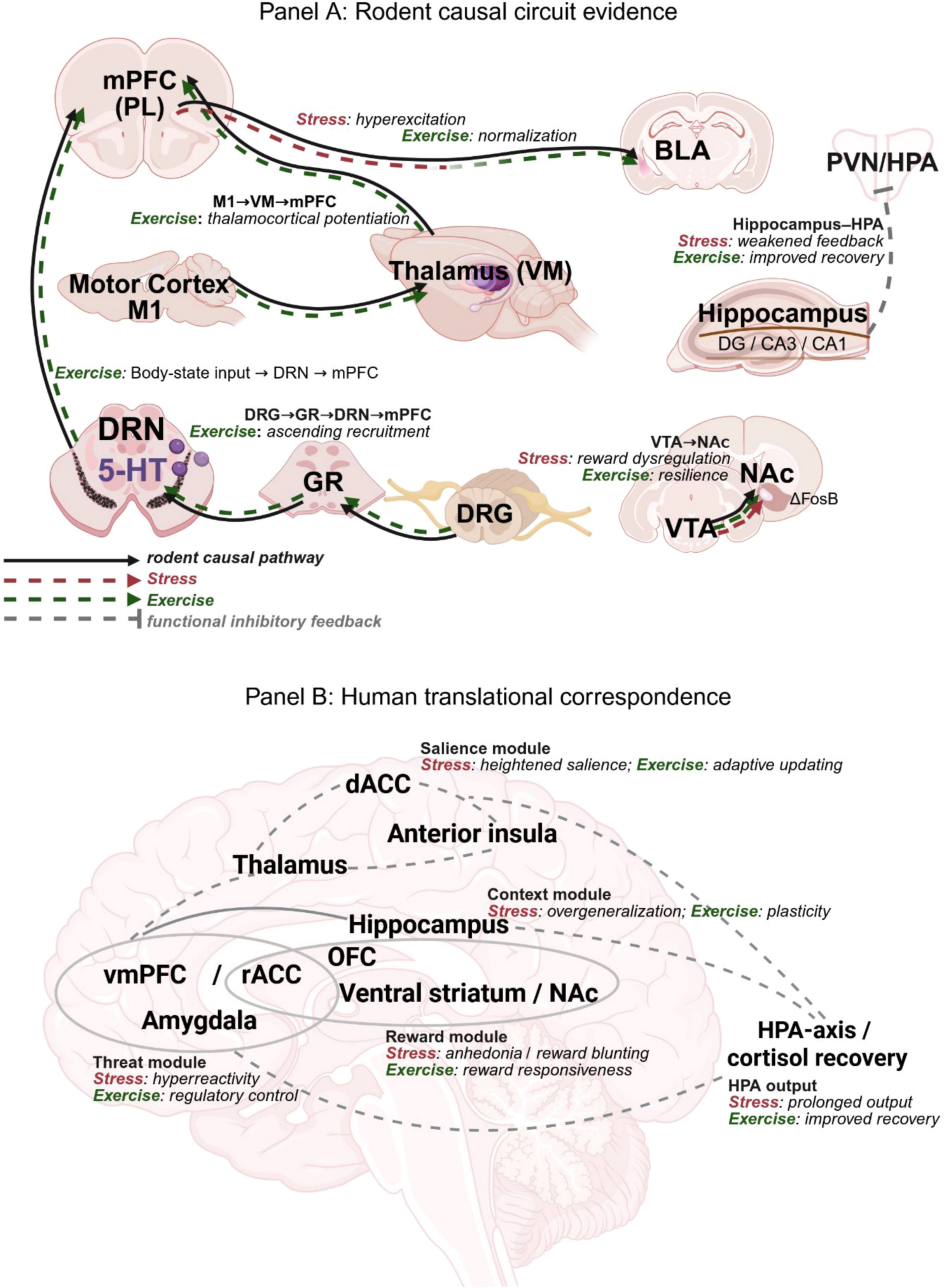
Exercise-modulated stress-regulation circuits across species. **(A)** Rodent causal circuit evidence. Black solid arrows indicate anatomically and functionally supported directional pathways emphasized in rodent studies. Red dashed arrows indicate stress-related dysregulation, and green dashed arrows indicate exercise-related normalization, potentiation, or recruitment. The panel highlights five principal modules discussed in the text: prefrontal–amygdala threat regulation, motor–thalamocortical control, ascending body-state signaling, hippocampal feedback to hypothalamic–pituitary–adrenal output, and ventral tegmental area–nucleus accumbens reward-resilience signaling. Gray dashed lines indicate functional inhibitory feedback rather than a direct monosynaptic tract. **(B)** Human translational correspondence. Gray solid and dashed lines indicate region-level functional correspondence and systems-level coupling derived primarily from human neuroimaging and fear-regulation literature, rather than validated monosynaptic projections. The panel summarizes five translational modules: prefrontal–amygdala threat regulation, hippocampus–prefrontal contextual control, salience-generalization coupling, reward valuation, and hypothalamic–pituitary–adrenal-axis/cortisol recovery output. Together, the two panels distinguish rodent causal circuitry from human network-level correspondence and provide a cross-species framework for interpreting exercise-related stress adaptation. ACC, anterior cingulate cortex; BLA, basolateral amygdala; dACC, dorsal anterior cingulate cortex; DRG, dorsal root ganglion; DRN, dorsal raphe nucleus; GR, gracile nucleus; HPA, hypothalamic–pituitary–adrenal; M1, primary motor cortex; mPFC, medial prefrontal cortex; NAc, nucleus accumbens; OFC, orbitofrontal cortex; PL, prelimbic cortex; rACC, rostral anterior cingulate cortex; VM, ventromedial thalamus; vmPFC, ventromedial prefrontal cortex; VTA, ventral tegmental area.

Chronic stress causes the brain to become biased towards hypervigilance and anhedonia. Exercise combats this by physically and functionally rewiring the specific neural circuits responsible for these processes. Figure 1 summarizes the principal circuits discussed in this section across two levels of evidence: Panel A highlights rodent causal circuit evidence, whereas Panel B summarizes the corresponding human translational networks. Table 1 summarizes the corresponding evidence base across circuits, readouts, and levels of causal support. In this section, mechanistic claims are drawn mainly from rodent studies unless human neuroimaging or clinical evidence is explicitly indicated. These circuits are relevant not only to broad anxiety- and depression-related phenotypes, but also to fear learning, fear extinction, and threat generalization, domains in which prefrontal inhibition of amygdala output, hippocampal contextual gating, and HPA-axis regulation are tightly coupled (Milad et al., 2006; Fullana et al., 2018). In Figure 1, Panel A therefore emphasizes rodent causal circuitry, whereas Panel B presents the corresponding human network-level correlates and translational convergence rather than direct monosynaptic pathway evidence.

**TABLE 1:** Evidence matrix for key stress and exercise neural circuits.

Circuit	Exercise	Stress	Causality	Species/model	References
mPFC → BLA	Treadmill normalizes mPFC–BLA function; anxiety-like **↓**	CRS recruits maladaptive top-down drive; anxiety-like ↑	Nec.	Mouse; CRS + treadmill 14 d	(Luo et al., 2023)
M1 → VM thalamus → mPFC (PrL)	Treadmill potentiates M1→VM→mPFC excitation; anxiety-like **↓**	CRS disrupts thalamo-cortical gating; anxiety-like ↑	Both	Mouse; CRS + treadmill 14 d	(Luo et al., 2025)
DRG → gracile nucleus → DRN (5-HT) → mPFC	Exercise engages hindbrain to forebrain chain; depressive-/anxiety-like **↓**	Stress model increases depression/anxiety-like ↑	Both	Mouse; exercise + stress-like phenotyping	(Lan et al., 2025)
Hypothalamus ↔ cerebellar dentate ↔ amygdala PKC**δ^+^**	Motor training engages anxiolytic loop; dentate to amygdala defined neurons	Motor challenge recruits loop engagement	Both	Mouse; CUMS 14 d + rotarod running 3 d	(Zhang et al., 2024)
LC galanin system → mesolimbic/cortex	Wheel running induces galanin; blocks stress neurochemical and structural effects	Stress: DA overflow ↑ + spine loss; vulnerability ↑	Nec.	Rat; wheel running 21 d + stress (footshock)	(Sciolino et al., 2015)
PVN→ NAc OT	Exercise restores PVN–NAc OT; antidepressant-like effects	Stress disrupts PVN–NAc OT; depressive-like ↑	Both	Mouse; CRS + treadmill 28 d	(Xia et al., n.d.)
NAc **Δ** FosB module	Wheel running induces NAc ΔFosB; CSDS resilience↑	CSDS differentiates susceptibility versus resilience	Causal/partial	Mouse; VWR 21 d + CSDS 10 d	(Mul et al., 2018)
Red nucleus (RNm) ↑ VTA glutamate	Exercise engages RNm↑VTA; reward relevant	Stress effect not primary focus	Both	Mouse	(He et al., 2022)

The Stress and Exercise columns indicate the dominant direction of change reported in the cited study. Causality summarizes the level of causal support (Nec., necessity; Both, necessity and sufficiency; Causal/partial, causal manipulation without full necessity and sufficiency). mPFC, medial prefrontal cortex; BLA, basolateral amygdala; M1, primary motor cortex; VM, ventromedial thalamus; PrL, prelimbic cortex; DRG, dorsal root ganglion; DRN, dorsal raphe nucleus; 5-HT, serotonin; vHPC, ventral hippocampus; LC, locus coeruleus; PVN, para-ventricular nucleus of the hypothalamus; NAc, nucleus accumbens; OT, oxytocin; BNST, bed nucleus of the stria terminalis; VTA, ventral tegmental area; CRS, chronic restraint stress; CUMS, chronic unpredictable mild stress; CSDS, chronic social defeat stress; VWR, voluntary wheel running.

### 3.1 Prefrontal amygdala circuits: restoring top-down control

Chronic stress biases this system toward persistent threat evaluation by weakening prefrontal control and amplifying amygdala output. However, exercise can counteract this shift by restoring prefrontal plasticity and strengthening inhibitory gating in the amygdala. At the molecular level, this circuit remodeling involves the coordinated epigenetic and translational regulation. For example, N^6^ methyl adenosine (m^6^A) RNA methylation shapes stress sensitivity by regulating the stability and translation of transcripts associated with plasticity (Engel et al., 2018). Meanwhile, exercise has been demonstrated to reduce stress-induced anxiety and alter brain RNA methylation profiles (Yan et al., 2022). The TrkB signaling pathway converts experience into protein synthesis, with the Phosphoinositide 3-kinase–protein kinase B–mTOR axis driving dendritic and synaptic restructuring (Zhang et al., 2014; Wu et al., 2024). Exercise can activate cortical Fragile X messenger ribonucleoprotein to mTOR signaling, thereby enhancing resilience and alleviating translational repression (Yan et al., 2023).

These molecular programs are relevant at the circuit-level because they regulate whether prefrontal neurons can maintain effective inhibitory control over amygdala output under stress. In this framework, m^6^A-dependent transcript regulation, TrkB-linked translational programs, and lactate-sensitive chromatin or protein modifications are not parallel observations, but candidate mechanisms through which exercise stabilizes synaptic efficacy, preserves excitation–inhibition balance, and restores top-down control within the prefrontal–amygdala circuit.

Furthermore, exercise links metabolic state to synaptic regulation through lactate-mediated lactylation. Histone lactylation establishes lactate as a substrate for the regulation of genes associated with chromatin (Zhang et al., 2019), while histone deacetylase (HDAC) 1–3 can remove lactyl marks, creating a dynamic and reversible system (Moreno-Yruela et al., 2022). Recent studies indicate that exercise increases the lactylation of multiple synaptic proteins in the medial prefrontal cortex (mPFC). Lactylation of synaptosome associated protein 91 is necessary for maintaining synaptic function and behavioral resilience under chronic stress (Yan et al., 2024). The circuit specificity is demonstrated by the discovery that the mPFC to basolateral amygdala pathway is critical for exercise-induced resilience (Luo et al., 2023, Figure 1).

In terms of mechanism, this recovery not only involves connectivity; it also involves the balance of excitation and inhibition. At the amygdala level, exercise training can reduce the S-nitrosylation level of gephyrin in the basolateral amygdala, increase surface expression of the γ-aminobutyric acid type A (GABA_*A*_) receptor γ2 subunit, and enhance the inhibitory effect, thus limiting the threat of transmission (Yang et al., 2024). In short, exercise promotes flexibility to respond to threats by restoring the plasticity of the prefrontal lobe and enhancing the inhibition control of the amygdala. This provides a multi-level framework for intervention in stress-related emotional disorders.

### 3.2 Thalamocortical and sensorimotor pathway: the body-brain loop

Exercise is not only a metabolic challenge; it also generates proprioceptive, vestibular, and interoceptive inputs. We propose that this sensory movement pathway provides a regulatory signal. It engages ascending pathways and reshapes anxiety-related networks. First, regular movement output appears to strengthen the “top-down” control. In mice exposed to chronic restraint stress, 14 days of treadmill training prevented anxiety-like behavior by potentiating a thalamocortical pathway linking primary motor cortex input, ventromedial thalamus, and the mPFC (Luo et al., 2025, Figure 1). Because treadmill running is externally imposed, we note that this standardized paradigm is not equivalent to voluntary running and may itself recruit stress-related responses (Svensson et al., 2016). Causal experiments confirm that the thalamocortical circuit is not only an observer, but also a necessary channel for movement to play a protective role. This finding provides a circuit-level explanation of how motor system activity can directly influence prefrontal regulation of threat perception.

The corticothalamic drive is accompanied by “bottom-up” sensory pathways, which can also regulate emotional state. A three-synaptic pathway has been described that links the periphery to the forebrain. It involves activation of neurons in the gracile nucleus by dorsal root ganglia inputs, recruitment of dorsal raphe serotonergic neurons. Eventually, this drives activity in pyramidal neurons in the mPFC (Lan et al., 2025). In mice, Lan et al. identified an exercise-responsive dorsal root ganglion–gracile nucleus–dorsal raphe–mPFC tri-synaptic pathway whose engagement was associated with improved anxiety-like readouts and enhanced pathway connectivity. These findings are better interpreted as changes in arousal and affect-related regulation, rather than as direct evidence for human-like negative emotion in rodents.

Together, these findings suggest that exercise appears to supply the brain with continuous remodeling signals that recalibrate the thalamic gating and update internal state estimation, thus reducing hypervigilance. These rodent circuit mechanisms provide a biological basis for the human translational networks summarized in Figure 1B, including prefrontal-limbic regulation, contextual control, salience-generalization coupling, and reward-related network adaptation (Phelps et al., 2004; Milad et al., 2005; Milad et al., 2009; Erickson et al., 2011; Morriss et al., 2018; Saanijoki et al., 2018; Webler et al., 2021; Won et al., 2023; Kommula et al., 2024). The focus of future translational research is to bridge this gap by combining specific circuit records of rodents with standardized behavioral paradigms. This will involve quantifying the impact of these ascending signals on safety learning and threat generalization.

### 3.3 Hippocampal plasticity: context, memory bias, and adult neurogenesis

The hippocampus acts as the brain’s contextual anchor, encoding the location and timing of experiences while also regulating the output of the HPA axis. This dual role is key to distinguishing actual threats and safe contexts. This regulatory influence over the HPA axis is mediated in part by hippocampal glucocorticoid receptor and mineralocorticoid receptor signaling. Under basal conditions, high-affinity mineralocorticoid receptors maintain tonic inhibitory tone over the HPA axis, while glucocorticoid receptors are recruited at peak glucocorticoid concentrations to terminate the stress response via negative feedback projections to the paraventricular nucleus of the hypothalamus (De Kloet et al., 1998; Herman et al., 2016). Chronic stress impairs this feedback by reducing hippocampal glucocorticoid receptor expression and cornu ammonis 1/3 (CA3) neuronal integrity, thereby weakening the brake on HPA axis output and sustaining glucocorticoid exposure—a self-reinforcing cycle that accelerates hippocampal damage.

However, chronic stress can weaken this precision. Structurally, pyramidal neurons in the CA3 region can show apical dendritic retraction, an early sign of circuit damage that worsens with prolonged stress (Woolley et al., 1990; Conrad et al., 2017). Functionally, pattern separation depends on the sparse coding in the dentate gyrus and the tightly regulated transmission to CA3 through mossy fibers (Bischofberger et al., 2006; Guzman et al., 2021). Stress-related remodeling in this region is also shaped by corticosteroid signaling at CA3 dendrites, further linking glucocorticoid exposure to impaired contextual discrimination and memory bias. Stress disrupts this fine-tuned regulation by reducing metabolic and trophic support, as reflected by downregulation of the BDNF/TrkB signaling and reduced GABAergic markers (Thompson Ray et al., 2011). Together, these changes provide a biological basis for negative memory bias and the tendency to overgeneralize threats to safety situations.

Exercise combats these stress-induced deficits by restoring plasticity in the hippocampus at both temporal and structural levels. Short-term exercise rapidly alters activation patterns in the dentate gyrus and CA3 regions. Exercise promotes hippocampal volume expansion and microstructural integrity, even in ageing populations (Creer et al., 2010; Vivar et al., 2016; Firth et al., 2018; Frodl et al., 2020; Callow et al., 2023). Mechanistically, exercise triggers presynaptic, phosphorylation-dependent remodeling in conjunction with BDNF/TrkB signaling. This process stabilizes synaptic transmission and resets plasticity thresholds. The co-localization of BDNF with synapsin I in CA3 and dentate gyrus suggests a targeted improvement in transmission fidelity rather than a general increase in excitability (Frodl et al., 2020). Critically, this restoration of hippocampal cellular integrity has direct implications for HPA axis regulation. By preserving glucocorticoid receptor-mediated negative feedback capacity in CA1 and CA3, exercise may help normalize the set point of the stress response, reducing the tendency toward glucocorticoid hypersecretion that characterizes chronic stress states (Godoy et al., 2018). Consistent with this, exercise training in rodent models attenuates stress-induced corticosterone elevations and accelerates post-stress recovery, effects that are partially dependent on intact hippocampal glucocorticoid receptor signaling (Agorastos and Chrousos, 2022).

Although adult hippocampal neurogenesis, which is enhanced by running and linked to improved pattern separation, contributes to these benefits, it is not essential for all gains induced by exercise (Creer et al., 2010; Anacker et al., 2018). A more comprehensive explanation is that exercise acts across multiple systems simultaneously: it increases trophic support, strengthens inhibitory control, boosts vascular and metabolic capacity, and enhances circuit gating. Beyond neurons, exercise also influences glial populations relevant to stress adaptation. Available evidence indicates that physical activity can dampen pro-inflammatory microglial activation, alter astrocytic support functions, and, in some contexts, promote oligodendrocyte maturation and myelin-related plasticity, although these mechanisms remain less well resolved in stress-specific paradigms (Maugeri et al., 2023; Yamaguchi et al., 2023). Neurogenesis serves as a context-dependent regulatory role in this integrated adaptive response (Schoenfeld et al., 2016).

Exercise may also influence fear-related learning more directly. In rodent models, acute exercise has been reported to facilitate fear extinction in some paradigms, including mTOR-dependent effects, although results are not uniformly positive across protocols (Jacquart et al., 2017; Moya et al., 2020). In humans, convergent fear-conditioning and neuroimaging studies identify mPFC, amygdalar, and hippocampal circuitry as key substrates of extinction recall (Fullana et al., 2018), and emerging work suggests that aerobic exercise after extinction learning may reduce subsequent threat expectancy (Crombie et al., 2023).

### 3.4 Mesocorticolimbic reward circuits: motivation, anhedonia, and stress coping

Exercise is also reinforced by the mesocorticolimbic reward system. The system focuses on dopaminergic projections from the ventral tegmental area (VTA) to the nucleus accumbens, assigning motivational value to environmental cues (Figure 1). Chronic stress can decouple effort and reward: the metabolic or psychological cost of an action no longer yields the expected hedonic or functional return. This manifests clinically as anhedonia and learned helplessness (Amat et al., 2006; Nestler and Carlezon, 2006).

Mechanistically, the VTA to the nucleus accumbens projection plays a key role in encoding both exercise motivation and emotional valence (Talalaenko et al., 1994; Pezze and Feldon, 2004; de Oliveira et al., 2009; Esch and Stefano, 2010). In mice with low baseline physical activity, chemogenetic activation of tyrosine hydroxylase positive neurons in the VTA promotes voluntary wheel running and enhances resilience to chronic social defeat stress (Zhang et al., 2021). Further downstream, it seems that exercise restores dopaminergic signaling by making coordinated changes to dopamine synthesis, reuptake and postsynaptic receptor sensitivity. This could involve increasing the amount of tyrosine hydroxylase, changing how the dopamine transporter works and making receptors more or less responsive. Table 1 summarizes the main circuits involved in stress and exercise effects regulation and highlights where causal evidence is still limited.

## 4 Molecular and cellular substrates: converting movement into synaptic change

Not all pathways discussed below occupy the same mechanistic tier. In the present framework, bioenergetic state and recovery capacity provide the permissive context for adaptation according to allostatic triage models (Shaulson et al., 2024; Kelley et al., 2025). Within this context, BDNF–TrkB signaling, mTOR- and AMP-activated protein kinase (AMPK)-linked translation control, and m^6^A-related proteomic tuning function as core integrative axes that determine whether exercise is converted into durable plasticity. By contrast, lactylation, neuromodulators, and many circulating exerkines more often act as modulatory or context-dependent contributors whose effects vary by timing, cell type, and regional state. Evidence strength and mechanistic tier are therefore related but not identical.

### 4.1 Neurotrophic signaling and synaptic remodeling

BDNF and its high-affinity receptor TrkB constitute the primary regulators of exercise-induced plasticity. While chronic stress typically triggers BDNF downregulation, leading to synaptic pruning and circuit fragility, exercise serves as a potent inducer of BDNF transcription, translation, and secretion. Although hippocampal BDNF responses are the most extensively studied, exercise-related BDNF/TrkB signaling is not restricted to the hippocampus and likely contributes to plasticity in prefrontal, mesolimbic, and other stress-relevant networks. The fundamental challenge of how the brain detects peripheral movement is supported by multiple metabolic messengers. For example, the lactate produced by exercise can cross the blood-brain barrier (BBB) via the monocarboxylate transporters. There, it triggers a transcription program that relies on sirtuin 1, which recruits the proliferator-activated receptor gamma coactivator 1-alpha (PGC-1α) to the BDNF promoter (El Hayek et al., 2019). This shows that there is a direct link between metabolism and gene regulation, so that the hippocampus can adjust its plasticity according to the energy consumption of the whole body.

Multiple secretory factors derived from the muscle and liver further enhance these central neurotrophic pathways. Endurance training induces muscle-specific PGC-1α/fibronectin type III domain containing 5 (FNDC5) pathways, thus releasing irisin into the blood. Peripheral FNDC5/irisin signaling has been reported to increase hippocampal BDNF, although the precise route by which circulating irisin accesses the brain remains under active investigation (Wrann et al., 2013). These peripheral signals do not work in isolation. They work synergistically with other cyclic factors to stabilize the TrkB signaling pathway and improve the accuracy of synaptic transmission in the prefrontal-hippocampal network (Lu et al., 2024). We caution against treating circulating BDNF as a direct surrogate of central plasticity, because blood BDNF reflects multiple peripheral sources, is strongly influenced by platelet release and pre-analytical handling, and does not map straightforwardly onto region-specific brain TrkB engagement. For translational studies, downstream markers such as TrkB phosphorylation, extracellular vesicle cargo, or multimodal imaging behavioral readouts may provide more interpretable evidence of central target engagement.

### 4.2 Epitranscriptomic regulation and translation control: m^6^A as a plasticity switch

Epitranscriptomic regulation, especially m^6^A modification, enables the brain to rapidly adjust its proteome in response to environmental challenges. Unlike slower genomic changes, m^6^A methylation of mRNA can act as a dynamic switch that controls transcript stability, localization and translation. In the adult brain, m^6^A signaling is sensitive to glucocorticoids. Chronic stress can disrupt this process, leading to dysregulation of key genes related to mPFC plasticity. Recent evidence suggests that m^6^A readers can promote protein synthesis during long-term potentiation. Crucially, these findings position the epitranscriptome layer as a gatekeeper of the plasticity threshold that supports effective stress adaptation (Engel et al., 2018; Shi et al., 2018).

The unique potential of exercise is that it can normalize stress-induced m^6^A dysregulation through a new metabolic axis from the liver to the brain. Emerging studies show that physical activity not only resets brain methylation through neural activity, but also achieves this goal by systematically mobilizing liver resources. Exercise can increase the production of methyl donor molecules in the liver. These molecules are then transported to the brain, where they support the restoration of healthy m^6^A patterns in the mPFC, thus effectively reversing anxiety-like behavior (Yan et al., 2022). However, the functional effect of this signaling fundamentally depends on its cellular environment. For instance, the alkylation repair homolog protein 5 demethylase shows heightened sensitivity to stress specifically within astrocytes. Research shows that cell type-specific manipulation of alkylation repair homolog protein 5 in mPFC can regulate depressive behavior in both directions by controlling the translation of glutamate transporter 1 (Guo et al., 2024). Looking to the future, this field must transition from holistic tissue analysis to cell type discrimination analysis. It should integrate these epitranscriptome changes with readings at the circuit level. This will help clarify how systemic metabolism determines the accuracy of threat appraisal and safe learning.

### 4.3 The role of mTOR and metabolic signals in stress circuits

mTOR, especially mTOR complex 1, is the main metabolic plasticity sensor of the brain. It converts systemic hormonal and nutritional signals into a translation program for precise timing. mTOR complex 1 combines the energy state of organisms with structural remodeling to determine the dendritic complexity and synaptic efficacy of the hippocampal and cortex circuits. Long-term exposure to stress and glucocorticoid environments will weaken the accuracy of this sensor, thus causing inhibition of mTOR signal transduction, which is harmful to organisms. This is manifested in the shrinkage of dendrites and the loss of plasticity (Polman et al., 2012). mTOR does not act as a static effector, but coordinates multi-level cell adaptation by integrating stress response transcription procedures to ensure that the plasticity of the brain matches the available metabolic resources (Aramburu et al., 2014). This metabolic emotional interface is especially obvious in the AMPK-mTOR-autophagy axis. Here, impaired nutrient sensing will cause the neural network to show anxiety-like behavior, directly linking systemic energy failure with psychological vulnerability (Li et al., 2022). This also implies that exercise-responsive mTOR signaling is permissive rather than self-sufficient: without adequate recovery and energetic support, activation of translational programs may fail to consolidate adaptation into durable network stability.

Exercise can dynamically regulate the mTOR program, reshape the energy flow of the system, and induce regular neural activity, thus stabilizing the network that is susceptible to stress. In the paraventricular nucleus of the hypothalamus, mTOR complex 1 integrates exercise-induced metabolic changes and environmental inputs, acts on corticotropin-releasing hormone neurons, effectively resets metabolic and emotional control (Hu et al., 2016). However, the therapeutic window for mTOR modulation is narrow. Recent biochemical evidence further emphasizes the important role between mTOR and integrated stress response by regulating the general control of nonderepressible 2 (Darawshi et al., 2024). This shows that the impact of exercise is not just about activating mTOR. On the contrary, it coordinates systematic metabolic resets and recalibrates the translation threshold across the brain. The challenge of future research is to clarify how exercise-induced mTOR signals can stably control specific resilience networks through cell type-specific interference.

### 4.4 Lactate, lactylation, and protein level rewiring

Lactate has moved beyond its traditional identity as a by-product of glycolysis and has become a metabolic-to-structure link connecting systemic physical activity with circuit remodeling. In addition to providing energy to neurons and inducing neurotrophic effects similar to BDNF, exercise-derived lactate can act as a signal that drives lysine lactylation. This post-translational modification links metabolic state to gene expression. Lactylation was first described on histones and is considered to be a chromatin regulatory mechanism. Recently, lactylation has been detected in brain tissue, and its levels fluctuate with neural activity and stress-related stimulation (Zhang et al., 2019; Hagihara et al., 2021). This suggests that lactate is not only a marker of metabolism, but also a dynamic sensor of physiological experience.

The functional significance of this metabolic signal is that it can reshape the synaptic structure, especially in the mPFC, thus stabilizing anxiety resistance. Research indicates that exercise-induced lactate surges increase the lactylation of multiple synaptic proteins; notably, lactylation of synaptosome associated protein 91 in the mPFC appears essential for maintaining synaptic integrity and buffering the effects of chronic stress (Yan et al., 2024). This process is highly regulated and reversible, controlled by the deacetylase activity of HDAC1-3, which provides a mechanism for the timing regulation of resilience-related gene programs (Moreno-Yruela et al., 2022).

However, the functional characteristics of lactylation fundamentally depend on the specific situation. In terms of exercise and stress tolerance, lactylation contributes to adaptive plasticity, but abnormal lactylation is associated with neuroinflammatory and neurodegenerative diseases. This highlights the critical importance of the timing, cell type and regional distribution of lactylation (Xiong et al., 2024; Qin et al., 2025). Future research must prioritize the mapping of lactylation of a specific loop by integrating the causal disturbances of regionally distinguished proteomics and monocarboxylate transporters. This will help clarify how these metabolic transients are consolidated into persistent safety and resilience. Thus, lactylation should not be considered beneficial in all cases. Its functional direction is likely shaped by dose, temporal pattern, cell type, and regional context.

### 4.5 Neuromodulators and the pleasure of runners: endocannabinoids, opioids, and monoamines

After endurance exercise, anxiety decreases and the pain threshold increases. It is commonly known as the runner’s high, which is a complex neurochemical adaptation. Although historically attributed to endogenous opioids (endorphins), the consensus has definitively shifted toward recognizing the endocannabinoid (eCB) system as the primary regulator of exercise-induced anxiolysis. This paradigm shift is supported by rigorous cross-species evidence. In mice, the anti-anxiety and analgesic effects of running are not eliminated by the opioid blockade, but are instead eliminated by the chemogenetic inhibition of the cannabinoid receptor 1 on the GABAergic neurons in the forebrain (Fuss et al., 2015). Similarly, in a double-blind, placebo-controlled study, blocking opioid receptors failed to reduce euphoria or anxiety after running, and circulating eCB levels were closely related to the improvement of these emotional states (Siebers et al., 2021).

However, the eCB hypothesis rests on a complex physiological background. Meta-analyses show that although acute exercise continues to increase circulating eCB levels, the degree of this increase varies widely (Desai et al., 2022). This variability may reflect circadian gating, because eCB signaling is regulated by circadian rhythms that also interact with stress and memory consolidation (Morena et al., 2022). In addition, eCBs do not act in isolation; on the contrary, they can function as a negative feedback mechanism activated by the HPA axis, linking exercise-induced affective changes with homeostatic buffering of the stress response (Riebe and Wotjak, 2011).

Acetylcholine is another relevant neuromodulator because cholinergic signaling shapes attention, hippocampal encoding, and fear-related plasticity. Outside canonical stress paradigms, recent work in a postoperative cognitive dysfunction model suggests that exercise-conditioned plasma can enhance hippocampal cholinergic circuit activity and acetylcholine release alongside BDNF/TrkB activation, indicating that cholinergic tone may represent one route by which exercise opens a plasticity window for adaptive learning (Lu et al., 2024).

Overall, exercise engages a coordinated set of neurotransmitters rather than a single molecule. The acute surge in eCBs, opioids, dopamine, and serotonin create a unique neuroplastic state. From a stress resilience perspective, the functional value of this state is not transient euphoria but the cognitive window it opens. By temporarily reducing anxiety and increasing reward sensitivity, exercise can support safety learning, fear extinction, and cognitive reappraisal. Thus, the runner’s high may prime the organism toward active coping rather than passive withdrawal.

## 5 Peripheral-to-brain mediators: myokines, metabolites, and immune–metabolic crosstalk

**FIGURE 2:**
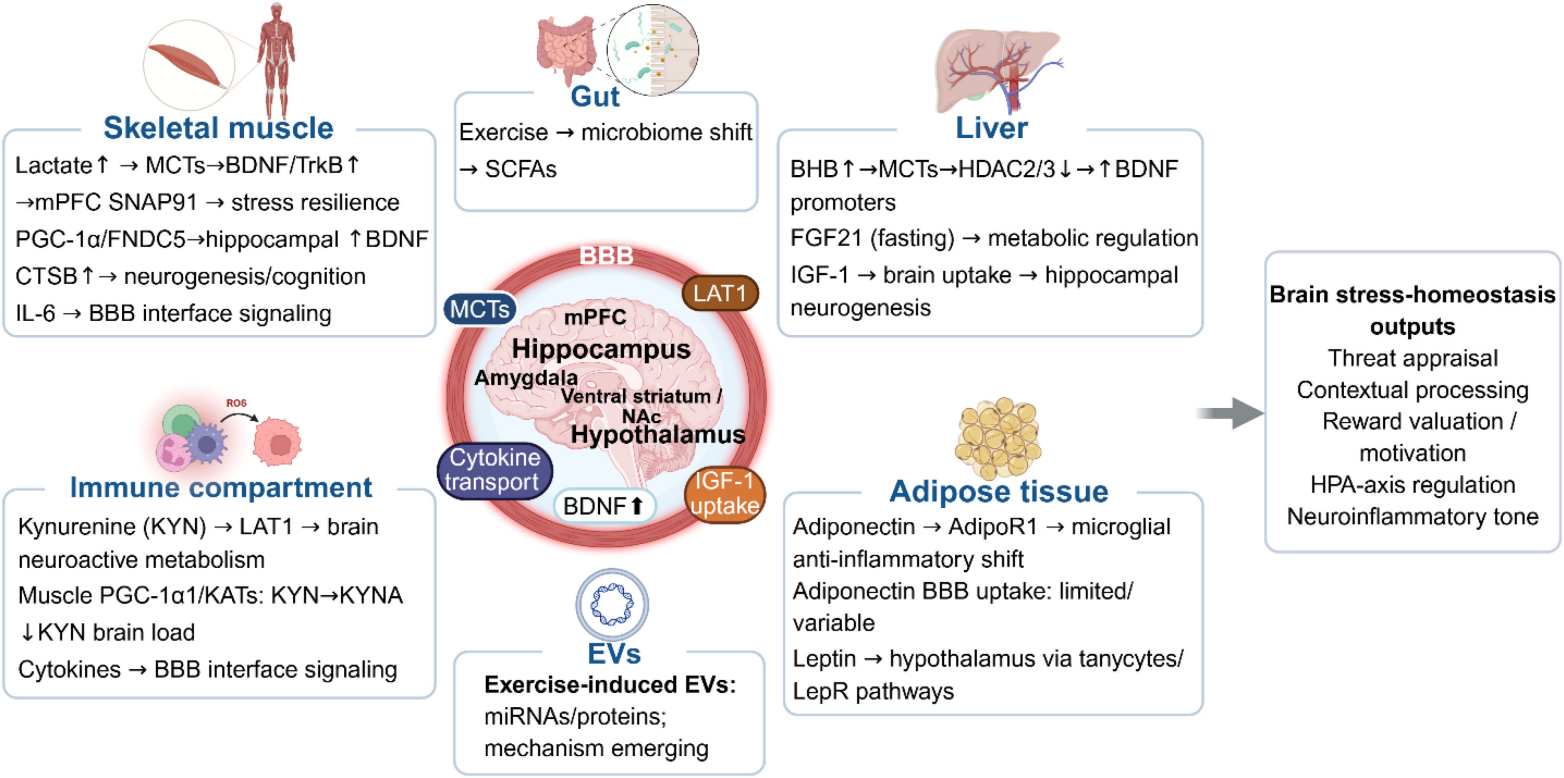
Multi-organ peripheral mediators and transport routes linking exercise to central stress regulation. Exercise remodels peripheral organs and systemic physiology, generating metabolites, hormones, cytokines, and extracellular vesicles that reach the brain through defined interface routes, including monocarboxylate transporters, large neutral amino acid transporter 1, cytokine and endothelial signaling at the blood–brain barrier, and related uptake or barrier mechanisms. The schematic summarizes candidate signals from skeletal muscle, gut, liver, adipose tissue, immune compartments, and extracellular vesicles that converge on stress-relevant brain regions, including medial prefrontal cortex, hippocampus, amygdala, hypothalamus, and reward-related forebrain circuits. The rightmost output layer indicates the principal dimensions of stress-related homeostatic regulation emphasized in this review, including threat appraisal, contextual processing, reward valuation/motivation, HPA-axis regulation, and neuroinflammatory tone. This figure is intended as an integrative functional map rather than a complete wiring diagram. BBB, blood–brain barrier; BHB, β-hydroxybutyrate; CTSB, cathepsin B; EVs, extracellular vesicles; HPA, hypothalamic–pituitary–adrenal; IGF-1, insulin-like growth factor 1; KATs, kynurenine aminotransferases; KYN, kynurenine; LAT1, large neutral amino acid transporter 1; MCTs, monocarboxylate transporters; mPFC, medial prefrontal cortex; SCFAs, short-chain fatty acids.

Exercise also reshapes peripheral organs and systemic physiology, which can influence the brain through circulating factors and barrier interfaces. We focus on four sources of circulating molecules: muscle, liver and vasculature, adipose tissue, and multi-source mediators that include gut, immune, and vesicle based signaling. Figure 2 summarizes these peripheral-to-brain routes, and Table 2 lists representative mediators with their evidence strength and proposed mechanisms. The relevance of these peripheral mediators to stress regulation lies not merely in their circulation, but in how they influence central processes that are stress-sensitive, including neuroinflammation, HPA-axis gating, reward valuation, contextual plasticity, and BBB signaling.

**TABLE 2:** Candidate peripheral mediators linking exercise to central stress regulation.

Mediator	Primary source	Brain uptake	Central targets	Evidence level
Skeletal muscle-derived mediators				
Lactate (El Hayek et al., 2019; Yan et al., 2024)	Skeletal muscle glycolysis; blood lactate rise	Established: MCT1/2 (blocked by AR-C155858)	Hippocampus (BDNF/TrkB); mPFC synapses (SNAP91 lactylation)	Both; MCT2, BDNF TrkB; SNAP91 lactylation
Cathepsin B (CTSB) (Moon et al., 2016; De la Rosa et al., 2019; Zheng et al., 2025)	Skeletal muscle myokine release with running	Unclear: circulating CTSB	Dentate gyrus neurogenesis; astrocyte reactivity	Nec.; dentate gyrus neurogenesis
FNDC5/Irisin (Wrann et al., 2013; Lourenco et al., 2019; Islam et al., 2021; Kim et al., 2023)	PGC-1α FNDC5 program; circulating irisin; hippocampal induction	Emerging: peripheral-to-brain signaling route under investigation	Hippocampal neurons; astrocytes	Both; PGC-1α FNDC5 BDNF
Kynurenine pathway shift (Agudelo et al., 2014; Walker et al., 2019; Minhas et al., 2024)	Muscle PGC-1α1 → KAT induction; KYN to KYNA conversion	Established: KYN influx via LAT1	Neurons, astrocytes	Causal partial; PGC-1α KAT kynurenine shunt
Liver/vascular-derived mediators				
β-hydroxybutyrate (BHB) (Pierre and Pellerin, 2005; Sleiman et al., 2016)	Liver ketogenesis (endurance, fasting, ketogenic states)	Established: ketones via MCT1/2	Hippocampal neurons; epigenetic BDNF regulation	Causal partial; HDAC2/3; BDNF
BHB–amino acids (Moya-Garzon et al., 2025)	Ketosis induced BHB shunt metabolites (CNDP2-dependent)	Unclear: CNS entry not fully resolved	Hypothalamus and brainstem neurons; appetite suppression	Both; CNDP2; BHB Phe; feeding circuits
IGF-1 (Trejo et al., 2001; Pristerà et al., 2019; Chen et al., 2025; Yu et al., 2025)	Liver endocrine; exercise promotes brain uptake	Established: activity-dependent uptake into brain regions	Neural progenitors; microglia–neuron IGF-1 axis; DA neurons	Nec.; IGF-1 uptake, adult neurogenesis
VEGF-A (Fabel et al., 2003; Deyama et al., 2020; Benevento et al., 2024)	Peripheral/vascular growth factor ↑ with exercise	Probable: neurovascular niche; (paracrine/endothelial)	Endothelium (vascular niche; neurons (VEGF–Flk-1); tanycytes (VEGF-A release)	Nec.; VEGF Flk1, neurovascular niche
Adipose-derived mediators				
Adiponectin (Spranger et al., 2006; Kusminski et al., 2007; Sun et al., 2019; Chen Z. et al., 2024; Liu et al., 2024)	Adipose endocrine; exercise may ↑ levels/sensitivity	Limited BBB entry; CNS effects shown via local receptor/circuit	Microglia state; VTA DA neurons (AdipoR1); BLA to PrL circuit	Nec.; AdipoR1 AMPK, microglia polarization
Leptin (Tan et al., 2024; Francois et al., 2025; Solheim et al., 2025)	Adipose endocrine; energy status signal	Established: saturable transport; tanycyte mediated uptake	Hypothalamic LepR neurons (ARC, DMH); tanycytes	Mech.; tanycyte LepR transport, energy balance
Multi-source/systemic mediators				
Short-chain fatty acids (SCFAs) (Braniste et al., 2014; Erny et al., 2015; Goswami et al., 2018; Tang et al., 2022; Forte et al., 2024)	Gut microbiota fermentation; diet/exercise reshape output	Probable: gut absorption; vagal afferents and FFAR2/3; limited BBB uptake	Microglia maturation; BBB endothelium; hypothalamic orexin neurons	Both; FFAR2, microglia maturation
Interleukin-6 (IL-6) (Banks et al., 1994; Willis et al., 2020; Sun et al., 2024)	Myokine cytokine ↑ acutely with exercise	Established: saturable BBB transport; endothelial signaling; CVOs (area postrema)	Area postrema neurons; BBB endothelium; microglia	Mech.; BBB transport, area postrema
Extracellular vesicles (EVs) (Antoniou et al., 2023; Delgado-Peraza et al., 2023; Cleary et al., 2025)	Multi-tissue release; exercise alters EV cargo	Probable: EV brain uptake; multiple vesicle populations reported	Neurovascular unit; microglia/astrocytes; neurons	Suff.; ExerVs cargo, adult neurogenesis
Clusterin (CLU) (De Miguel et al., 2021; Chen C. et al., 2024)	Systemic plasma factor ↑ with exercise/aging interventions	Circulating protein; BBB interface signaling at neurovascular/immune sites	Endothelium/BBB; microglia (complement), astrocytes (context-dependent)	Both; complement inhibition, neuroinflammation

Key peripheral metabolites, hormones, cytokines, and extracellular vesicle–associated signals are summarized with their primary source tissues, brain uptake or BBB interface routes, central targets, and evidence level. Evidence level (last column): Nec. necessity (loss of function/blockade); Suff. sufficiency (gain of function/delivery); Mech. Brain relevant access/interface evidence only; Causal partial causal support for a pathway component only; Both. necessity and sufficiency. AMPK, AMP-activated protein kinase; ARC, arcuate nucleus; AR-C155858, MCT1/2 inhibitor; BBB, blood–brain barrier; BDNF, brain-derived neurotrophic factor; BHB, β-hydroxybutyrate; BLA, basolateral amygdala; CLU, clusterin; CNDP2, carnosine dipeptidase 2; CTSB, cathepsin B; DA, dopamine/dopaminergic; DMH, dorsomedial hypothalamus; EVs, extra-cellular vesicles; ExerVs, exercise-derived EVs; FNDC5, fibronectin type III domain-containing protein 5; HDAC2/3, histone deacetylase 2/3; IGF-1, insulin-like growth factor 1; IL-6, interleukin-6; KAT, kynurenine aminotransferase; KYN, kynurenine; KYNA, kynurenic acid; LepR, leptin receptor; MCT1/2, monocarboxylate transporter 1/2; mPFC, medial prefrontal cortex; PGC-1α1, peroxisome proliferator-activated receptor gamma coactivator 1-alpha isoform 1; PrL, prelimbic cortex; SCFAs, short-chain fatty acids; SNAP91, synaptosome associated protein 91; TrkB, tropo-myosin receptor kinase B; VEGF-A, vascular endothelial growth factor A; Flk-1, VEGF receptor 2; VTA, ventral tegmental area.

### 5.1 The contracting muscle secretome: from neurotrophic drivers to metabolic detoxification

Skeletal muscle is no longer viewed merely as a mechanical motor, but rather as the body’s largest endocrine organ. During contraction, skeletal muscle releases hundreds of myokines and metabolites collectively termed exerkines, establishing a molecular dialogue with the brain. This communication is primarily regulated by the transcription coactivator PGC-1α, which not only remodels muscle metabolic mechanisms but also induces the secretion of factors capable of crossing the BBB to modulate central plasticity. In this framework, Table 2 highlights four muscle linked candidates with possible peripheral-to-brain routes, including lactate, cathepsin B, FNDC5 irisin signaling, and exercise induced kynurenine pathway remodeling.

Neurotrophic Drivers: irisin and cathepsin B. Endurance training activates a specific PGC-1α-dependent pathway that drives the expression of FNDC5, a membrane protein subsequently cleaved to form the circulating myokine irisin (Figure 2, muscle-derived mediators). Irisin has been identified as a candidate peripheral mediator of exercise-induced brain plasticity. Peripheral FNDC5/irisin signaling has been reported to increase hippocampal BDNF and support neuroprotective responses, although the precise route by which circulating irisin accesses the brain remains under active investigation (El Hayek et al., 2019; Lourenco et al., 2019). Operating in parallel is cathepsin B, a lysosomal protease whose secretion is upregulated during running. In contrast to inflammatory cytokines, muscle-derived cathepsin B is beneficial. It can pass through the BBB to enhance hippocampal neurogenesis and spatial memory (Moon et al., 2016), which highlights a non-classical role of protease in inter-organ communication.

Peripheral clearance: In addition to releasing factors that promote plasticity, skeletal muscles also provide tolerance by metabolizing and detoxifying kynurenine. Chronic stress can increase the level of kynurenine in the circulation. Kynurenine is a metabolite of tryptophan, which easily enters the brain and produces neurotoxic by-products related to depressive-like phenotype. Adaptive skeletal muscle can be used as a peripheral removal route for kynurenine. Exercise-induced PGC-1α upregulates the expression of kynurenine aminotransferases. These enzymes can convert kynurenine into kynurenic acid, which is less able to cross the BBB (Agudelo et al., 2014). Muscles effectively protect the brain from neurotoxic effects caused by stress by converting kynurenine into a harmless form and isolating it from the periphery.

Although a single myogenic factor shows prospects, exercise releases a complex mixture of signals, including lactate and various exosomes. One of the main limitations of the current research is that it is difficult to distinguish the causal relationship in the system environment. Stronger inferences need to go beyond correlation and turn to muscle-specific gene regulation and adequacy testing using homoprotein delivery. In addition, clarifying the muscle-brain axis will require separating the direct effects of myokines from their indirect interactions with immune signaling, including the context-dependent anti-inflammatory actions of exercise-induced interleukin-6 (IL-6). These muscle-to-brain signals are therefore relevant to stress management not simply because they rise with exercise, but because they can bias central systems toward trophic support, lower neurotoxic load, and improved resilience to chronic stress exposure.

### 5.2 Liver and vascular-derived mediators: β-hydroxybutyrate, BHB–amino acids, IGF-1, and VEGF-A

Liver ketogenesis and vascular growth signaling provide a major peripheral route by which exercise can affect neural plasticity in the brain. Table 2 highlights four candidates with mechanistic links to central adaptation, including β-hydroxybutyrate (BHB), BHB to amino acid conjugates, circulating insulin-like growth factor 1(IGF-1), and vascular endothelial growth factor A (VEGF-A; Figure 2, liver-derived mediators).

BHB is a liver-derived ketone that rises during prolonged exercise and enters brain via monocarboxylate transporters (Pierre and Pellerin, 2005). In mice, BHB promotes hippocampal BDNF programs by inhibiting HDAC2 and HDAC3 at selective genomic regions, increasing BDNF promoter activity and BDNF expression, consistent with BHB acting as an instructive metabolic signal rather than a passive fuel (Sleiman et al., 2016).

Ketone signaling may also extend beyond BHB itself. A recently described β-hydroxybutyrate shunt pathway generates BHB–amino acid conjugates, suggesting that ketosis can diversify its circulating outputs; however, the relevance of these metabolites to exercise-related stress resilience remains indirect and is not yet established in canonical stress paradigms (Moya-Garzon et al., 2025).

Complementing its systemic endocrine functions, IGF-1 serves dynamic roles through localized brain signaling. Beyond the classical finding that circulating IGF-1 fuels exercise-driven neurogenesis (Trejo et al., 2001), specific neural populations utilize distinct reservoirs of the protein. Microglia-associated signaling governs developmental growth and stress resilience. More recent studies implicate microglia-related IGF-1 signaling in developmental neurogenesis and stress resilience phenotypes (Chen et al., 2025; Yu et al., 2025), while dopamine neuron-derived IGF-1 actively tunes neuronal firing to support learning and exploration. Therefore, there is no single mechanism (Pristerà et al., 2019). Instead, it involves a flexible inter-play between peripheral inflow and local cell sources, depending on the context.

VEGF-A links vascular adaptation to neural plasticity. Peripheral VEGF is necessary for running induced adult hippocampal neurogenesis (Fabel et al., 2003), and neuron specific VEGF shapes synaptic and cognitive function (Deyama et al., 2020). VEGF-A also supports plasticity at hypothalamic vascular interfaces, which may regulate how circulating state signals access feeding and stress control circuits. More broadly, recent circuit work outside exercise-specific stress paradigms highlights the hypothalamus as an interface that converts body-state cues into motivated behavior (Benevento et al., 2024). Collectively, these observations suggest that vascular signaling and hypothalamic gating may shape how peripheral metabolic states are translated into brain outcomes, although direct links to exercise-related stress adaptation remain to be established. In this framework, liver and vascular-derived signals contribute to stress regulation insofar as they shape the metabolic reserve and neurovascular plasticity that permit stress-related circuits to adapt rather than destabilize.

### 5.3 Adipose-derived mediators: adiponectin and leptin

Adiponectin is an adipokine with anti-inflammatory and insulin sensitizing properties that links peripheral metabolic state to stress-relevant brain processes. In rodent stress models, running reduces hippocampal neuroinflammation and shifts microglia toward an anti-inflammatory state through adiponectin/adiponectin receptor 1 signaling, while adiponectin can also modulate VTA dopamine neuron activity and anxiety-related behavior (Sun et al., 2019; Liu et al., 2024). Mechanistically, adiponectin does not readily cross the BBB, but it can influence brain endothelium and is detectable in cerebrospinal fluid in distinct complexes (Spranger et al., 2006; Kusminski et al., 2007).

Leptin is included here primarily as a broader metabolic-state signal rather than as a stress-specific exercise mediator. It tunes hypothalamic and extra-hypothalamic circuits involved in feeding, thermogenesis, and motivated behavior, but the current circuit literature is not exercise specific and does not directly establish a role in exercise-related stress resilience (Tan et al., 2024; Solheim et al., 2025). Accordingly, leptin is best viewed as contextual endocrine background that may shape stress responsiveness indirectly, pending studies that combine peripheral leptin perturbation with standardized exercise and stress phenotyping.

### 5.4 Multi-sources systemic mediators: SCFAs, IL-6, extracellular vesicles, and clusterin

Some exercise relevant signals arise from multiple sources and act as system-level state cues rather than organ specific messengers. Four candidates with accumulating mechanistic support are short-chain fatty acids (SCFAs), IL-6, extracellular vesicles (EVs), and clusterin (CLU).

SCFAs provide a gut-derived route to neuroimmune and hypothalamic regulation. The microbiota continuously shapes microglial maturation and function, establishing a baseline pathway by which peripheral ecology can tune central immune tone (Erny et al., 2015). SCFAs also engage fast neural access points by activating vagal afferent neurons and can modulate orexin or hypocretin neurons that regulate arousal and motivated behavior (Goswami et al., 2018; Forte et al., 2024). In metabolic stress models, SCFAs have been reported to rescue hippocampal neurogenesis and BBB integrity while improving depressive-like behavior (Tang et al., 2022). Because microbiota can influence BBB permeability more broadly, barrier gating is likely a key intermediate phenotype that will vary across individuals (Braniste et al., 2014).

IL-6 is especially context dependent. During exercise, transient peripheral IL-6 elevations can function as part of an adaptive myokine response, whereas sustained central or systemic IL-6 elevation is more often associated with chronic neuroinflammatory burden (Funk et al., 2011). Animal studies further suggest that exercise can reduce stress- or disease-associated microglial activation and IL-6-related inflammatory signaling in the brain (Kohman et al., 2013), while IL-6 can also participate in repair programs under selected conditions (Willis et al., 2020). We therefore interpret IL-6 as a timing and compartment-sensitive mediator rather than as uniformly harmful or beneficial.

EVs provide a packaging mechanism for coordinated molecular cargo that can be tracked peripherally and may have functional effects. Neuron derived EVs are being developed as peripheral readouts of brain signaling states, including insulin related dysregulation in neurodegenerative disease (Cleary et al., 2025), and exercise alters neuron derived EV signatures in Alzheimer’s disease cohorts (Delgado-Peraza et al., 2023). Mechanistically, neuronal EV-associated microRNAs can induce circuit connectivity downstream of BDNF, supporting plausibility of EVs as effectors rather than only biomarkers (Antoniou et al., 2023). The key unresolved issue is whether specific EV cargo is sufficient to transfer stress resilience phenotypes across individuals.

CLU provides one of the clearest examples of a transferable plasma factor linked to brain anti-inflammatory effects. Exercise enhanced memory and reduced neuroinflammatory responses through the circulation system, and CLU was implicated as a candidate mediator of this systemic anti-inflammatory signature (De Miguel et al., 2021). However, CLU biology is source and context dependent. Astrocyte derived CLU can disrupt glial physiology and obstruct remyelination in demyelinating disease models, cautioning against unidirectional interpretations (Chen C. et al., 2024). For exercise-related stress regulation, CLU is therefore best evaluated together with complement pathway and microglial state readouts. A consolidated overview of candidate peripheral mediators, their potential routes to the brain, and the current level of evidence is provided in Table 2.

## 6 Future directions

### 6.1 From activity to dosimetry: standardizing the physiological stimulus

A common limitation in the current literature is the simplification of physical activity into binary behavioral variables (e.g., active versus sedentary), rather than treating it as a gradable physiological intervention. Due to significant variations in exercise intensity and stress protocols across rodent studies, coupled with the influence of baseline health status and participant compliance heterogeneity in human trials, dose determination remains imprecise, resulting in a substantial translational gap in research findings. To elevate exercise to the status of precision medicine, future research must transition from defining dose by external work (e.g., speed or duration) to clamping intensity against internal biological markers, such as lactate threshold or heart rate reserve. By reporting core physiological parameters and standardized stress stimulation readings, this field can ensure that different subjects experience equivalent metabolic stress, so as to achieve repeatable cross-study comparisons. However, internal load matching alone is insufficient. The same workload may fall within a hormetic zone in one individual but exceed adaptive capacity in another (Mattson and Leak, 2024). Precision exercise should therefore incorporate recovery kinetics, metabolic flexibility, and bioenergetic reserve in addition to real-time physiological intensity markers.

### 6.2 Causality, cross-species connection, and personalization

Establishing the effectiveness of the mechanism requires a rigorous translation from describing the phenomenological relationship to proving the causal relationship. For peripheral mediators, this means going beyond the correlation test and turning to the tests of necessity and sufficiency. Accordingly, acute changes in catecholamines, endocannabinoids, lactate, or mood should be interpreted as state markers, whereas sustained changes in HPA recovery, downstream trophic signaling, circuit properties, and behavior measured beyond the immediate post-exercise window are more consistent with consolidation. At the same time, in order to bridge the differences in species, the research results at the rodent circuit level must be associated with non-invasive readouts in humans. Therefore, this field must acknowledge and address individual differences. Future research should classify individuals not only by baseline metabolism or genetic characteristics, but also by baseline stress responsivity, cumulative prior exposure, recovery opportunity, and metabolic reserve. This shift from dose matching to adaptive-capacity matching may help explain why similar exercise prescriptions produce durable recalibration in some individuals but only transient perturbation or maladaptation in others.

## 7 Discussion

As a controllable systemic challenge, exercise can recalibrate stress-related homeostasis, but only when repeated exposures are coupled to adequate recovery and bioenergetic support. Here, recalibration refers to a durable shift in the operating range of stress-responsive systems rather than a transient neuromodulatory state induced by a single bout. It includes more appropriate HPA-axis reactivity and recovery, restored circuit signal-to-noise and excitation–inhibition balance, more adaptive plasticity thresholds for contextual and reward processing, and sufficient metabolic reserve to stabilize these changes over time. It is reflected in refined prefrontal-amygdala regulation, improved hippocampal contextual processing, and restored mesocorticolimbic motivational drive. Core molecular mediators include the BDNF-TrkB axis, mTOR-mediated translation control, and m^6^A epitranscriptomic remodeling. These mediators couple synaptic function to metabolism through mechanisms such as lactate-linked lysine lactylation, translating transient metabolic states into the longer-lasting synaptic resilience. To translate these insights into practice, the field must prioritize exercise dose definition and causal validation. Fundamentally, exercise represents a systematic reset, which supports adaptive capacity and strengthens physical activity as a practical intervention under stress. This hormetic framing also suggests an upper limit: excessive training load, long-term recovery deficits, or pre-existing energy limitations may convert exercise from a recalibrating stimulus into an additional stress burden (Mattson and Leak, 2024). In practice, this should be reflected not only in reduced symptom burden, but also in improved fear discrimination and extinction, more appropriate HPA-axis recovery, and reduced threat overgeneralization across contexts.
